# Supplementary material for: Learning curve and surgical outcome of robotic assisted colorectal surgery with ERAS program
Source: Sci Rep. 2022 Nov 29;12:20566. doi: 10.1038/s41598-022-24665-w (PMC9709162; doi:10.1038/s41598-022-24665-w)
Supplement: Supplementary file 2 — Supplementary Information 2. [file 41598_2022_24665_MOESM2_ESM.zip › RAL Raw data-Ñ[▒K/130/OP N_ñw╝╨░O▒Kñσ.pdf]

W95 / W95

臺中榮民總醫院
手術室護理記錄

[redacted]

基本資料

- 室別： 開刀房-17室，手術科別： CRS，手術類別： 常規，日期： 2021/12/02
- 進入時間： 1055 ； 麻醉開始時間： 1058 ； Time Out時間： 1150 ； 劃刀時間： 1150 ； 手術結束時間： 1710 送出時間： 1735 ；
- 麻醉方式： EA
  診斷：Malignant tumor of sigmoid colon
  手術名稱：Robot Assisted Colectomy(L)
  手術局部用藥：NIL
  術前預防性抗生素：
  - 抗生素名稱一： Veterin 1GM IVD由麻醉護理人員協助執行
  - 時間： 1135
  - 上次簽章: 蘇敬涵

  ，追加預防性抗生素：
  - 抗生素名稱一： Veterin 1GM IVD由麻醉護理人員協助執行
  - 時間： 1535
  - 上次簽章: 鄭鈞躍
- 手術醫師： 蔣鋒帆，陳瑋夫，蔡濬樟
- 麻醉醫師： VS 沈靜慧
-
  - 刷手護士A ： 歐真勤，時間： 1055，接替刷手一： 羅媗鈴/廖子晴，時間： 1130，接替刷手二： 歐真勤/蘇敬涵，時間： 1500
  - 流動護士A ： 蘇敬涵，時間： 1055，接替流動一： 蘇敬涵/林貝臻，時間： 1130，接替流動二： 鄭鈞躍，時間： 1500

皮膚狀況

A-V Shunt： 無
術前： 完整
記錄者： 上次簽章: 蘇敬涵
術後： 完整
記錄者： 上次簽章: 鄭鈞躍

手術準備

手術臥式 單一手術臥式

-
  - 手術臥式： 截石臥式
  - 使用附件： 矽膠枕頭，腳架，其它： 廠商翻身用物
  - 皮膚準備： 10%Aq Betadine，Hibiscrub，2% Chlorhexidine
  - 電燒： 使用電燒: 電極片位置： 其它： 迴路電擊墊，記錄者： 上次簽章: 蘇敬涵

止血帶T： 無

植入物

- Davinci Stapler 45 Green x2/Colon

標本送檢

- 細菌培養 無
- 細胞學 無
- 冰凍切片 無
- 病理標本
  組織名稱一：S colon
  ,
  組織名稱二：IMA root
  ,

組織名稱三： Distal cut end

- 截肢 無

標本交付 標本： 1 袋 ， 標本： 0 盒 ， 標本： 2 瓶 ， 細胞學： 0 管 ， Culture Tube： 0 管 ， 被交付醫師： R 蔡濬樟 ，

- 記錄者： 上次簽章: 鄭鈞躍
- 時間： 1630

傷口術後敷料

傷口敷料： 傷口敷料： 無

- 請於圖上標示部位：

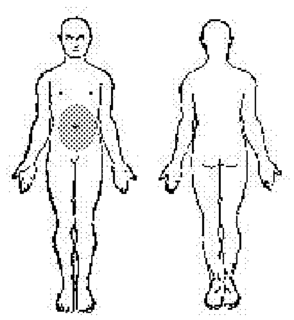

腹部TROCAR洞

- 傷口敷料： Mepore

導管

導管	天數	導管資訊	插入日期	插入時間	更換日期	拔除日期	拔除時間	拔除原因	預計拔除/更換	預計拔除/更換日期
Foley(1)	1	號碼:16 Fr.; 部位:膀胱;	2021/12/02	1110						
Sump NG Tube(1)	1	號碼:16 Fr.; 深度:55cm; 部位:胃;	2021/12/02	1130						
Double Lumen CVC(1)	1	號碼:8 Fr.; 深度:15cm; 部位:jugular (右); 象限:RU;	2021/12/02	1120						
A-line (1)	1	號碼:20 G; 部位:左手;	2021/12/02	1115						

用物計數

- 手術中出血量： minimal c.c.
- 手術中尿量： 500 c.c.
-
    - 其它： 無

用物計數： 多切口同時縫合

- 1.器械： 正確，2.縫針： 正確，3.刀片： 正確，4.紗布： 正確，5.腹部墊： 未使用，6.紗球： 未使用，7.PEANUTS ： 未使用，8.紗條： 未使用，9.絲帶： 未使用，10.針頭： 未使用，11.Vessel loop： 未使用，12.Boots： 未使用，13.Jelco： 未使用，14.棉條： 未使用，
    - 第一次：
        - 刷手護士 ： 歐真勤
        - 流動護士： 上次簽章: 鄭鈞躍
        - 時間： 1600

  ,
    - 第二次：
        - 刷手護士 ： 歐真勤
        - 流動護士： 上次簽章: 鄭鈞躍
        - 時間： 1620

  ,
    - 第三次：
        - 刷手護士 ： 歐真勤
        - 流動護士： 上次簽章: 鄭鈞躍

- 時間： 1640

備註

備註： Davinci 30度Scope NO.2

- 記錄者： 上次簽章: 蘇敬涵
- 時間： 1150

填寫護理人員_鄭鈞躍

正式摘要

W95 / W95

doctorRank:B
